# Preparing for the Impact of COVID-19 on the Mental Health of Youth

**DOI:** 10.1177/1942602X211052626

**Published:** 2022-03

**Authors:** Eileen R. O’Shea, Kathryn E. Phillips, Kathleen N. O’Shea, Linda N. Roney

**Affiliations:** Professor of Pediatric Nursing, Marion Peckham Egan School of Nursing & Health Studies, Fairfield University, Fairfield, CT; Associate Professor, Marion Peckham Egan School of Nursing and Health Studies, Fairfield, CT; Medical Intensive Care Unit Nurse, Boston Children’s Hospital, Boston, MA; Associate Professor, Marion Peckham Egan School of Nursing and Health Studies, Fairfield, CT

**Keywords:** youth, mental and behavioral health disorders, impact of COVID-19, at-risk behaviors, screening, assessment tools, suicide prevention

## Abstract

The COVID-19 pandemic is continuing to have long-term and global effects that the vaccine may not ease. Children and adolescents endured unprecedented periods of loneliness, social isolation, financial stressors, in-home conflicts, changes in living circumstances, and variable access to healthcare, resulting in increased mental health sequelae. Timely recognition of students’ anxiety, depression, and disruptive behaviors will allow appropriate interventions to de-escalate these feelings and prevent suicidal ideations and attempts. As youth return to school, their mental health needs will not subside. School nurses and the multidisciplinary team have a vital role in impacting this population’s already surging increase of mental and behavioral health disorders.

School nurses are often the first to identify students’ behavioral health and wellness concerns and connect families to community resources (National Association of School Nurses [NASN], 2021). In March 2020, in response to the COVID-19 pandemic, large-scale school closures ranged from 1 month to the duration of the academic year that limited students and families access to their school’s nurse (Rothstein & Olympia, 2020). While overall pediatric emergency department (ED) visits were down during the pandemic, the proportion of ED visits for mental health conditions significantly increased (Leeb et al., 2020; Yard et al., 2021). These pediatric patients were more likely to require admission and have more extended stays than patients before the pandemic (Krass et al., 2021).

In May 2020, shortly after the beginning of the pandemic, a Gallup Poll reported that 29% of school-age parents stated their child’s mental or emotional health was harmed (Calderon, 2020). By October 2020, another national survey conducted by the Jed Foundation reported that 31% of parents indicated their child’s emotional or mental health was worse than before the start of the pandemic (Panchal et al., 2021).

More specific findings have been reported from a national poll conducted among 977 parents with at least one child age 13- to 18 years old, which indicated the most common pandemic-related mental health conditions were depression and anxiety (C.S. Mott Children’s Hospital, 2021). The parents identified differences for adolescent females and boys, in which teen girls experienced an increase in anxiety/worry (36%) versus 19% in teen boys. Depression/sadness was reported more frequently by the parents of teen girls than the parents of teen boys (31% vs. 18%). Findings concerning other conditions had a more similar distribution among females and male teens: (a) sleep issues occurred in 24% (females) versus 21% (males), (b) withdrawal from family occurred in 14% (females) versus 13% (males), and (c) aggressive behaviors occurred 9% (females) versus 8% (males).

Furthermore, Thompson et al. (2021) reported suicide attempts (SA) and suicidal ideation (SI) in a sample of adolescents (age 11-18 years) psychiatrically hospitalized during COVID-19 showed SA and SI ratings were higher during this pandemic in comparison with the prior year. Specifically, COVID-related SI was linked to stressors related to missing special events, financial problems, in-home conflict, and changes in living circumstances (Thompson et al., 2021).

This early data resulting from the pandemic compounds an already dire state of the increasing prevalence of mental and behavioral health disorders within the pediatric and adolescent populations. Current statistics for the rate of suicide are now reported as the second leading cause of death in the United States for ages 10 to 24 years (Centers for Disease Control and Prevention, 2019). According to the 2019 Youth Risk Behavior Survey, one in five youths (18.8%) had seriously considered attempting suicide; one in six (15.7%) had made a suicide plan; one in 11 (8.9%) attempted; and one in 40 (2.5%) made an attempt that necessitated medical intervention (Ivey-Stephenson et al., 2020). In addition, prevalence rates were higher among sexual minority youths, those identified as lesbian, gay, or bisexual, and youths who reported having had sexual contact with the same or with both sexes (Ivey-Stephenson et al., 2020). Of utmost concern, the impact of COVID-19 and its mental health effects among the youth has yet to be fully understood. The concurrent factors of a public health crisis, social isolation, financial hardships, and healthcare access inequities may continue to affect the pediatric and adolescent population for years to come (Golberstein et al., 2020; Panchal et al., 2021).

In addition to the above data, clinicians must be mindful of the impact of the pandemic on normal developmental stages for the pediatric and adolescent populations. The school environment has a significant effect on youth’s development and relationships. Daily peer interactions for school-age children allow for the decreasing of egocentric perspectives as they navigate group pressures and become sensitive to social norms (Hockenberry et al., 2019). Likewise, during the adolescent phase of development, teens strive to become autonomous and self-reliant. The school environment provides opportunities to become emotionally independent from parents, allowing for relationship building of community networks outside of families (Hockenberry et al., 2019). In a divergence from the typical pre-COVID environment, youth have experienced “a prolonged state of physical isolation from peers, teachers, extended families, and community networks” (Loades et al., 2020, p. 1218).

With varying models of returning to in-person instruction implemented across the United States during the 2020-2021 academic year, the length of loneliness and social isolation thrust on students due to these disease mitigating efforts were also wide-ranging (Loades et al., 2020). Alarmingly, prior research suggests that social isolation and loneliness increase the risk of depression and possibly anxiety among children and adolescents.

## Assessing At-Risk Behaviors: What the School Nurse Needs to Know

Leaders in the field refer to the current state of growing behavioral and mental health needs in pediatrics as an epidemic (McMillan et al., 2019). As children return to school, nurses and the multidisciplinary team will be on the frontline of this epidemic. Early recognition and intervention of the most common pediatric mental health concerns being anxiety, depression, and disruptive behavior (Andrews et al., 2020), are critical in preventing the development of severe depression (Petito et al., 2020). In addition, in providing common at-risk behaviors and screening assessment tools, school nurses can feel confident in their role as an advocate and care coordinator to ensure students’ health and safety needs (NASN, 2016).

Given the prevalence of suicide among children and adolescents, it is essential to understand the risk and protective factors to help identify those who may have greater vulnerability to suicidality. A 2020 (Carballo et al., 2020) review of 44 studies on suicidality in children and adolescents found the following factors to be risks for suicidality: impulsivity, neuroticism, mood disorders, especially depressive disorders, substance misuse, prior suicidal behavior, family or peer conflicts. One challenge in risk identification has been distinguishing between those who experience SI and those who attempt suicide. Recent research (Mars et al., 2019) suggests that adolescents who had been exposed to self-harm in those close to them (a friend or family member) and those who had a psychiatric disorder were more likely to attempt suicide.

## Screening Children and Teens for Mental and Behavioral Health Disorders

In addition to recognizing at-risk behaviors, school nurses and the multidisciplinary team must be alert to pertinent data, including signs and symptoms, that may warrant further assessment of mental and behavioral health disorders. Utilizing assessment techniques, administering evidence-based instruments, collecting and documenting data can promote early identification of patterns and variances (American Nurses Association [ANA], 2015). Implementing mental health–screening tools for each school setting will require prior planning and approval from school administration and collaboration among the allied health professional team, such as school counselors, social workers, and psychologists. Establishing community relationships to effect safe transitions within the healthcare continuum is critical (ANA, 2015). Early identification will ensure timely referrals for further evaluation, reducing the risk of adverse health outcomes and promoting optimal functioning (ANA, 2015; Andrews et al., 2020). Table 1 lists the signs and symptoms along with many free validated measurement tools (see online supplemental file for embedded links) that may be utilized within school settings for the identification of common mental health disorders, including anxiety, depression, disruptive behavior, and suicidality (Andrews et al., 2020).

**Table 1.** Screening Children and Teens for Mental and Behavioral Health Disorders

Mental health disorders	Signs and symptoms	Assessment tools
Anxiety	Physical symptomsaRapid heart rateQuick breathing or difficulty catching one’s breathMuscle aches (especially stomach and headaches)Shaking, dizziness, tinglingSweatingFatigueEmotional symptomsaOngoing worries about friends, school, or activitiesWorrying about things before they happenA need for everything to be “perfect”Constant thoughts and fears about safety (of self or of others, such as parents and siblings)Reluctance or refusal to go to school“Clingy” behavior with parentsInability to concentrateIrritabilityTrouble sleepingInability to relax	Children’s Yale-Brown Obsessive Compulsive Scale (CY-BOCS) (Ages 6-17yrs)b Generalized Anxiety Disorder-7 (GAD-7) (Ages 12-18+yrs)b Penn State Worry Questionnaire for Children (PSWQ-C) (Ages 7-17yrs)b Revised Children’s Anxiety and Depression Scale (RCADS) (Grades 3-12)b Screen for Child Anxiety Related Emotion Disorders (SCARED) (Ages 8-18yrs)b Spence Children’s Anxiety Scale (SCAS) (Ages 8-18yrs)b
Depression	Core symptomsapersistent sadnesspersistent loss of interest in almost all activitiesAssociated symptomsaloss of energyloss of appetite (or increase)changes in sleeping patternsagitation or irritabilityfeelings of worthlessness or excessive guiltindecisivenesswanting to die	Center for Epidemiologic Studies Depression Scale for Children (CES-DC) (Grades 4-12)b Columbia Depression Scale (CDS; formerly DISC Depression Scale) (Ages 11-18+ yrs)b Depression Self-Rating Scale for Children (DSRSC) (Ages 8-18yrs)b Kutcher Adolescent Depression Scale (KADS) (Ages 12-17yrs)b Patient Health Questionnaire Depression Screeners (PHQ-9, PHQ-2) (Ages 13-18+yrs)b PHQ-9 Modified for Teens (Ages 11-17yrs)b Mood and Feelings Questionnaire (MFQ) (Ages 6-18+yrs)b
Disruptive Behavior	Oppositional defiant disordera persistent pattern of angry outbursts, arguments and disobedience. directed at authority figures, like parents and teachers, it can also target siblings, classmates and other children. Conduct disordera can involve cruelty to animals and people, other violent behaviors and criminal activity.	ADHD Rating Scale-IV (ADHD-RS-IV (Ages 6-15yrs)b Child and Adolescent Disruptive Behavior Inventory (CADBI) Screener (Ages 3-16yrs)b Disruptive Behavior Disorder Rating Scale (DBDRS) (Ages 5-10yrs)b Impairment Rating Scales (IRS) (Ages 4-12yrs)b NICHQ Vanderbilt Assessment Scales (Ages 5-15yrs)b Modified Overt Aggression Scale (MOAS) (Ages 6-18+yrs)b Overt Aggression Scale (OAS) (Ages 5-11yrs)b Swanson, Nolan, and Pelham rating scale (SNAP-IV) (Ages 6-18yrs)b Strengths and Weaknesses of ADHD symptoms and Normal behavior (SWAN) (Ages 3-18yrs)b
Suicidality	**Symptoms**a preoccupation with death (e.g., recurring themes of death or self-destruction in artwork or written assignments) intense sadness and/or hopelessness not caring about activities that used to matter social withdrawal from family, friends, sports, or social activities substance abuse sleep disturbance (either not sleeping or staying awake all night) giving away possessions risky behavior lack of energy inability to think clearly or problems with concentration declining school performance or increased absences from school increased irritability changes in appetite	Columbia-Suicide Severity Rating Scale (C-SSRS) (Ages 5-18+yrs)b Ask Suicide-Screening Questions (ASQ) (Ages 8-18yrs)c

a Boston Children’s Hospital Website: https://www.childrenshospital.org/disorders-and-treatments

b Adapted from: Andrews, Cho, Tugendrajch, Marriott, & Hawley, 2020

c National Institute of Mental Health: https://www.nimh.nih.gov/research/research-conducted-at-nimh/asq-toolkit-materials

## Mental Health Interventions

School nurses serve in many roles, such as healthcare providers, health educators, and care coordinators for those with mental health concerns (NASN, 2016). For parents, school nurses can provide referrals to local supports and be a point of contact to manage care between the home and school environment (Baker et al., 2017). For students, school nurses can intervene with those needing mental health support by utilizing motivational interviewing (MI), a commonly used and well-researched intervention in the school setting (Best et al., 2018). Allowing the student to be a partner in their care, MI is an open communication style rooted in empathy and supportive of self-efficacy (Beckwith & Beckwith, 2020). In the context of mental health concerns, the school nurse can use MI to enhance behavioral change or to have a collaborative conversation with students or their parents.

To provide comprehensive care, the school nurse can work with the interdisciplinary team, including the school social worker, guidance counselor, teachers, and school psychologist, to provide care to identified students through screening or frequent visits to the nurses’ office. Caring for some students can be time-consuming given the complexity of their conditions and the large caseload many nurses have (Ravenna & Cleaver, 2016). It may be helpful to have regular meetings with the interdisciplinary team to discuss cases and ensure optimal, coordinated care is provided to students in need.

The school nurse and interdisciplinary team can implement programming that builds skills to prevent mental illness and manage stressors at the school level. School-based mental health interventions are generally defined as either targeted (delivered to a specific group of students who are at risk) or universal (delivered to all students). A review of programs to impact anxiety and depression in school settings indicated that targeted and universal programs were valuable for students (Werner-Seidler et al., 2017). Meanwhile, universally delivered resilience-based school programs to improve mental health have been shown to be effective in children and adolescents (Dray et al., 2017; Fenwick-Smith et al., 2018). For suicide prevention, many programs have been developed and implemented. However, evidence of their effectiveness in preventing suicidal behavior is limited. Only a few programs show promising outcomes in randomized controlled trials (Katz et al., 2013), with a meta-analysis of studies showing that treatment effects were minor (Fox et al., 2021). In a 2013 review (Katz et al., 2013), two programs had evidence that they reduced SA: the Good Behavior Game, a skills training program, and Signs of Suicide, an awareness and education program. A review assessing the role of school nurses in suicide prevention programs found they are often not recognized as part of the mental health team (Pestaner et al., 2021). School nurses need to take a leadership role as a member of the school mental health team and have their contribution to the health of the student population recognized.

One challenge with providing mental health support is that school nurses often lack training in mental health and report a lack of confidence in this area (Ravenna & Cleaver, 2016). However, training in mental health improved confidence (Ravenna & Cleaver, 2016). Working with the interdisciplinary team, the school nurse can help to coordinate speakers and training that enhance all school staff’s understanding of mental health in the pediatric population. Additionally, providing community mental health agencies and resources for school staff may enhance their well-being and help ensure school staff can support students at this challenging time. Providing training and supporting staff are the two recommendations that The National Academies of Sciences, Engineering, and Medicine (2021) advise for supporting school staff in their recent publication, “School-based strategies to support the mental health and well-being of youth in the wake of COVID-19.” School nurses are positioned to be leaders in implementing mental health interventions that support individual students, groups, and the school community.

## Conclusion

As the new academic year begins with variability in vaccination rates, novel COVID-19 variants, and other significant uncertainties, it is clear that 2021-2022 will not provide an opportunity for return to our prepandemic state at our country’s schools. Therefore, student behavioral health and wellness must be prioritized for students to succeed in the academic setting (NASN, 2021) now more than ever.■
