# Supplementary material for: Preparing for the Impact of COVID-19 on the Mental Health of Youth
Source: NASN Sch Nurse. 2021 Dec 10;37(2):64–9. doi: 10.1177/1942602X211052626 (PMC8668437; doi:10.1177/1942602X211052626)
Supplement: Supplementary material [file sj-pdf-1-nas-10.1177_1942602X211052626.pdf]

Table 1. Screening Children and Teens for Mental and Behavioral Health Disorders

Mental Health Disorders	Signs & Symptoms	Assessment Tools
**Anxiety**	**Physical symptoms**[a]<br>Rapid heart rate<br>Quick breathing or difficulty catching one's breath<br>Muscle aches (especially stomach and headaches)<br>Shaking, dizziness, tingling<br>Sweating<br>Fatigue<br><br>**Emotional symptoms**[a]<br>Ongoing worries about friends, school, or activities<br>Worrying about things before they happen<br>A need for everything to be "perfect"<br>Constant thoughts and fears about safety (of self or of others, such as parents and siblings)<br>Reluctance or refusal to go to school<br>"Clingy" behavior with parents<br>Inability to concentrate<br>Irritability<br>Trouble sleeping<br>Inability to relax	Children's Yale-Brown Obsessive Compulsive Scale (CY-BOCS) (Ages 6-17yrs)[b]<br>https://iocdf.org/professionals/training-institute/btti/pediatric-materials/<br><br>Generalized Anxiety Disorder-7 (GAD-7) (Ages 12-18^{+}yrs)[b]<br>https://www.phqscreeners.com/<br><br>Penn State Worry Questionnaire for Children (PSWQ-C) (Ages 7-17yrs)[b]<br>https://www.childfirst.ucla.edu/resources/<br><br>Revised Children's Anxiety and Depression Scale (RCADS) (Grades 3-12)[b]<br>https://www.childfirst.ucla.edu/resources/<br><br>Screen for Child Anxiety Related Emotion Disorders (SCARED) (Ages 8-18yrs)[b]<br>https://www.pediatricbipolar.pitt.edu/resources/instruments<br><br>Spence Children's Anxiety Scale (SCAS) (Ages 8-18yrs)[b]<br>https://www.scaswebsite.com/

**Depression**	**Core symptoms**[a]<br>persistent sadness<br>persistent loss of interest in almost all activities<br><br>**Associated symptoms**[a]<br>loss of energy<br>loss of appetite (or increase)<br>changes in sleeping patterns<br>agitation or irritability<br>feelings of worthlessness or excessive guilt<br>indecisiveness<br>wanting to die	Center for Epidemiologic Studies Depression Scale for Children (CES-DC) (Grades 4-12)[b]<br>https://www.brightfutures.org/mentalhealth/pdf/tools.html<br><br>Columbia Depression Scale (CDS; formerly DISC Depression Scale) (Ages 11-18^{+} yrs)[b]<br>https://www.mdaap.org/pdf/Bi_Ped_CDSteen.pdf<br><br>Depression Self-Rating Scale for Children (DSRSC) (Ages 8-18yrs)[b]<br>https://www.childrenandwar.org/wp-content/uploads/2019/06/DSRSIN<br><br>Kutcher Adolescent Depression Scale (KADS) (Ages 12-17yrs)[b]<br>http://www.shared-care.ca/toolkits-mood<br><br>Patient Health Questionnaire Depression Screeners (PHQ-9, PHQ-2) (Ages 13-18^{+}yrs)[b]<br>http://www.cqaimh.org/pdf/tool_phq2.pdf<br><br>PHQ-9 Modified for Teens (Ages 11-17yrs)[b]<br>http://www.gladpc.org/<br><br>Mood and Feelings Questionnaire (MFQ) (Ages 6-18^{+}yrs)[b]<br>https://devepi.duhs.duke.edu/measures/the-mood-and-feelings-questionnaire-mfq/

**Disruptive Behavior**	**Oppositional defiant disorder**[a] persistent pattern of angry outbursts, arguments and disobedience. directed at authority figures, like parents and teachers, it can also target siblings, classmates and other children.<br><br>**Conduct disorder**[a] can involve cruelty to animals and people, other violent behaviors and criminal activity.	ADHD Rating Scale-IV (ADHD-RS-IV (Ages 6-15yrs)[b] https://pcptoolkit.beaconhealthoptions.com/wp-content/uploads/2016/01/cms-quality-child_adhd_rating_scale_screener.pdf<br><br>Child and Adolescent Disruptive Behavior Inventory (CADBI) Screener (Ages 3-16yrs)[b] https://osf.io/4j9gu/<br><br>Disruptive Behavior Disorder Rating Scale (DBDRS) (Ages 5-10yrs)[b] https://ccf.fiu.edu/research/_assets/dbd-rating-scale.pdf<br><br>Impairment Rating Scales (IRS) (Ages 4-12yrs)[b] https://ccf.fiu.edu/research/#online-resources<br><br>NICHQ Vanderbilt Assessment Scales (Ages 5-15yrs)[b] https://www.nichq.org/sites/default/files/resource-file/NICHQ-Vanderbilt-Assessment-Scales.pdf<br><br>Modified Overt Aggression Scale (MOAS) (Ages 6-18^{+}yrs)[b] https://depts.washington.edu/dbpeds/Screening%20Tools/Modified-Overt-Aggression-Scale-MOAS.pdf<br><br>Overt Aggression Scale (OAS) (Ages 5-11yrs)[b] https://www.researchgate.net/publication/19220225_The_Overt_Aggression_Scale_for_the_Objective_Rating_of_Verbal_and_Physical_Aggression<br><br>Swanson, Nolan, and Pelham rating scale (SNAP-IV) (Ages 6-18yrs)[b] http://www.shared-care.ca/toolkits-adhd<br><br>Strengths and Weaknesses of ADHD symptoms and Normal behavior (SWAN) (Ages 3-18yrs)[b]

		https://www.ncbi.nlm.nih.gov/pmc/articles/PMC4671522/

**Suicidality**	**Symptoms**[a]<br>preoccupation with death (e.g., recurring themes of death or self-destruction in artwork or written assignments)<br><br>intense sadness and/or hopelessness<br><br>not caring about activities that used to matter<br><br>social withdrawal from family, friends, sports, or social activities<br><br>substance abuse<br><br>sleep disturbance (either not sleeping or staying awake all night)<br><br>giving away possessions<br><br>risky behavior<br><br>lack of energy<br><br>inability to think clearly or problems with concentration<br><br>declining school performance or increased absences from school<br><br>increased irritability<br><br>changes in appetite	Columbia-Suicide Severity Rating Scale (C-SSRS) (Ages 5-18^{+}yrs)[b]<br>https://suicidepreventionlifeline.org/wp-content/uploads/2016/09/Suicide-Risk-Assessment-C-SSRS-Lifeline-Version-2014.pdf<br><br>Ask Suicide-Screening Questions (ASQ) (Ages 8-18yrs)[c]<br>https://www.nimh.nih.gov/sites/default/files/documents/research/research-conducted-at-nimh/asq-toolkit-materials/asq-tool/screening_tool_asq_nimh_toolkit.pdf

[a]Boston Children's Hospital Website: https://www.childrenshospital.org/disorders-and-treatments
[b]Adapted from: Andrews, Cho, Tugendrajch, Marriott, & Hawley, 2020
[c]National Institute of Mental Health: https://www.nimh.nih.gov/research/research-conducted-at-nimh/asq-toolkit-materials
